# Partitioning of beta‐diversity reveals distinct assembly mechanisms of plant and soil microbial communities in response to nitrogen enrichment

**DOI:** 10.1002/ece3.9016

**Published:** 2022-06-17

**Authors:** Weixing Liu, Xian Yang, Lin Jiang, Lulu Guo, Yaru Chen, Sen Yang, Lingli Liu

**Affiliations:** ^1^ State Key Laboratory of Vegetation and Environmental Change, Institute of Botany Chinese Academy of Sciences Beijing China; ^2^ University of Chinese Academy of Sciences Beijing China; ^3^ State Key Laboratory of Biocontrol, School of Ecology Sun Yat‐sen University Guangzhou China; ^4^ School of Biological Sciences, Georgia Institute of Technology Atlanta Georgia USA

**Keywords:** community assembly, community dissimilarity, N deposition, nestedness, partitioning *β*‐diversity, replacement, turnover

## Abstract

Nitrogen (N) deposition poses a serious threat to terrestrial biodiversity and alters plant and soil microbial community composition. Species turnover and nestedness reflect the underlying mechanisms of variations in community composition. However, it remains unclear how species turnover and nestedness contribute to different responses of taxonomic groups (plants and soil microbes) to N enrichment. Here, based on a 13‐year consecutive multi‐level N addition experiment in a semiarid steppe, we partitioned community *β*‐diversity into species turnover and nestedness components and explored how and why plant and microbial communities reorganize via these two processes following N enrichment. We found that plant, soil bacterial, and fungal *β*‐diversity increased, but their two components showed different patterns with increasing N input. Plant *β*‐diversity was mainly driven by species turnover under lower N input but by nestedness under higher N input, which may be due to a reduction in forb species, with low tolerance to soil Mn^2+^, with increasing N input. However, turnover was the main contributor to differences in soil bacterial and fungal communities with increasing N input, indicating the phenomenon of microbial taxa replacement. The turnover of bacteria increased greatly whereas that of fungi remained within a narrow range with increasing N input. We further found that the increased soil Mn^2+^ concentration was the best predictor for increasing nestedness of plant communities under higher N input, whereas increasing N availability and acidification together contributed to the turnover of bacterial communities. However, environmental factors could explain neither fungal turnover nor nestedness. Our findings reflect two different pathways of community changes in plants, soil bacteria, and fungi, as well as their distinct community assembly in response to N enrichment. Disentangling the turnover and nestedness of plant and microbial *β*‐diversity would have important implications for understanding plant–soil microbe interactions and seeking conservation strategies for maintaining regional diversity.

## Graphical abstract

With increasing N input, plant communities are increasingly driven by nestedness, fungal communities remain largely regulated by turnover and the turnover remains within a narrow range, whereas the profound turnover of bacteria increases greatly to contribute to the increased beta‐diversity of bacterial communities. These contrasting patterns indicate differences in the assembly mechanism of plant and soil microbial communities, with important implications for understanding aboveground‐belowground interactions under anthropogenic N deposition.

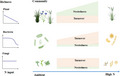

## 1 INTRODUCTION

Biodiversity is the foundation of ecosystem services which are closely related to human well‐being (Mori et al., 2018). Besides the well‐documented changes in the number of species at a locality (i.e., α‐diversity), changes in community composition, often quantified by beta (*β*)‐diversity, is another important metric of biodiversity change in response to environmental changes (Dornelas et al., 2014; Socolar et al., 2016). Exploring the changes and driving factors of *β*‐diversity could, therefore, provide important insights into the mechanisms underlying biodiversity change and the assembly of ecological communities in the face of environmental changes (Mori et al., 2018). Anthropogenic nitrogen (N) deposition is a major component of global change and one of the primary drivers of biodiversity loss worldwide (Stevens et al., 2004). There is increasing evidence that N enrichment alters plants (Tilman et al., 2004; Zhang et al., 2019) and soil microbial communities (Leff et al., 2015) in terrestrial ecosystems. Therefore, exploring the driving mechanisms of these communities under N enrichment could improve our understanding of changes in local biodiversity (Socolar et al., 2016). However, the mechanisms of community assembly of plant and soil microbial communities under N enrichment are, hitherto, underexplored.

Changes in community composition in responses to environmental changes (i.e., *β*‐diversity) could reflect two different phenomena of community re‐organization: species turnover and nestedness (Baselga, 2010; Williams, 1996). Species turnover indicates the difference in community composition caused by species replacement (Legendre, 2014), which encompasses the gain and loss of species after environmental changes (Leprieur et al., 2009). Conversely, nestedness indicates that one community with lower richness is a subset of the other with higher richness (Carvalho et al., 2013; Leprieur et al., 2011; Ulrich & Almeida‐Neto, 2012). It represents the difference in community composition caused by non‐random species loss and is usually accompanied by changes in species richness (Baselga, 2010; Ulrich & Almeida‐Neto, 2012). N enrichment could change community composition by promoting species turnover or nestedness. For example, N enrichment would enhance soil N availability and induce soil acidification. This change may act as an environmental filter that excludes some residents (Leigh et al., 2019) and initiates species turnover when some opportunistic species colonize the space vacated by the locally extinct species. Alternatively, nestedness would occur when environmental changes directly induce loss of species (Ulrich et al., 2009). Therefore, partitioning *β*‐diversity into species turnover and nestedness could reveal the processes and mechanisms governing community reassembly under N enrichment.

An important unanswered question is whether species turnover and nestedness of different taxonomic groups (e.g., plants, soil bacteria, and soil fungi) respond to N enrichment differently due to their different intrinsic physiological and metabolic properties (Mori et al., 2018; Norfolk et al., 2015; Schiel, 2019). It has been reported that species turnover is the main contributor to the *β*‐diversity of plants (Antao et al., 2019; Soininen et al., 2018). However, N enrichment stimulates plant growth and productivity, potentially resulting in competition for light and consequent species extinction (Harpole & Tilman, 2007). Changes in plant community composition, in this situation, would be mainly derived from nestedness, reflecting the loss of species. For soil microbial communities, changes in community composition might follow plant community shift and present similar patterns under N enrichment since plants provide substrates for soil microbial growth (Compant et al., 2010; Zheng & Gong, 2019). While soil microbial communities could show distinct patterns from plant communities because of strong dispersal ability and short generation times. First, microorganisms with smaller individual sizes and greater abundance have a greater probability of dispersal than plants (Zhou & Ning, 2017). Higher dispersal facilitates local microbial taxa to track suitable habitats in heterogeneous environmental conditions (Gianuca et al., 2017; Leibold et al., 2004), decreasing the possibility of taxa loss and consequent nestedness under N enrichment. Second, compared with plants, microbial populations are characterized by shorter life spans and faster growth (Shade et al., 2012). Microbial communities, therefore, might exhibit rapid species turnover to adapt to N enrichment. However, there is no evidence as to how and to what extent N influences turnover or nestedness components of plant and soil microbial communities under N enrichment.

Here, we undertook the first attempt to investigate the community compositional changes in plants, soil bacteria, and fungi under N enrichment while disentangling species turnover and nestedness components. The study was conducted based on manipulative N addition experiment (from 0 to 64 g N m^−2^) in a semiarid steppe. The atmospheric N deposition is up to 14.7 kg N hm^−2^ and is expected to continually increase in this area (Zhang et al., 2017). Plant and microbial communities have changed under extra N input, but the underlying mechanisms of community assembly are still unclear (Ling et al., 2017; Liu et al., 2020; Yao et al., 2014). We analyzed dissimilarity in community composition (i.e., *β*‐diversity) and partitioned their two components between communities with ambient N deposition and those with different levels of N addition, and aimed to answer two questions: (1) How species turnover and nestedness components contribute to changes in plant and microbial communities with increasing N input? (2) Which environmental variables drive the variation in the dominant *β*‐diversity components of plant and soil microbial communities?

## 2 MATERIALS AND METHODS

### 2.1 Study area and experimental design

The study was conducted based on a long‐term N addition platform at Duolun Restoration Ecology Research Station of the Institute of Botany (42°02′N, 116°17′E, and 1324 m.a.s.l.), Chinese Academy of Sciences in Duolun County, Inner Mongolia, China. The climate in this study area is cold semiarid, and mean annual temperature and precipitation are 2.1°C and 382.3 mm, respectively. The vegetation is steppe with a predominance of grasses (*Stipa krylovii*, *Cleistogenes squarrosa*, *Agropyron cristatum*) and forbs (*Artemisia frigida*, *Potentilla bifurca*). The soil type is classified as Haplic Calcisols according to Food and Agriculture Organization of the United Nations FAO classification. Soil organic C and total N contents are 16.94 and 1.65 g kg^−1^. Soil pH is approximately 6.84.

The N addition manipulative experiment was established in a native grassland with evenly distributed vegetation in 2003. The field experiment was set up with a Latin square design containing eight levels of N addition with eight replicates (layout with eight rows and eight columns, Figure S1). Four rows (one in every two adjacent rows) were clipped annually at the end of the growing season from 2005. Therefore, there were four blocks with a total of 32 non‐clipping plots (10 × 15 m) including eight levels of N addition. Eight levels of N addition 0, 1, 2, 4, 8, 16, 32, and 64 g N m^−2^ were applied in July each year (four replicates of each treatment across the experiment). The N addition levels were comparable to those N addition gradient experiments in other grasslands (Bai et al., 2010). We intentionally chose these N addition levels to establish a large N gradient to mimic high N deposition that is projected to occur in the future (Ramirez et al., 2010). All 10 × 15 m plots were separated by 5‐m buffer strips. We fertilized with Urea‐N due to the purchase restriction on ammonium nitrate by the Chinese government.

### 2.2 Sample collection and measurements

All plant and soil samples were collected from the non‐clipping plots. Plant community was investigated in August 2016 to determine species richness using the point intercept method. Briefly, a 1 × 1 m quadrat was randomly placed in each non‐clipping plot. The quadrat frame consists of 100 evenly distributed grids. We identified all plant species within those 100 grids in each plot. Soil sampling was carried out in non‐clipping plots in mid‐August 2016. Specifically, soil samples were taken randomly from the top 15 cm layer using a soil core with 5 cm diameter in each plot. The six cores were mixed thoroughly into one sample in each plot. Soil samples were passed through a 2 mm sieve to remove roots and stones and immediately transported to the lab. During transportation, we placed soil samples in cool boxes filled with blue ice packs. A subsample set was stored at ‐80°C for molecular measurement, and the others were stored at 4°C for soil physicochemical properties.

Fresh soil subsamples were used for soil inorganic N (DIN) extraction with 2 M KCl solution. The analysis of NH_4_^+^–N and NO_3_–N concentrations in solution was performed on a flow injection analyzer (SAN‐System, Breda, Netherlands). Soil elements and pH were measured using air‐dried subsamples. The details of measurement of soil‐extractable Al^3+^and Mn^2+^, soil N concentration, and soil pH value were described in a previous study by Liu et al., (2021).

### 2.3 Molecular method and bioinformatic analysis

We extracted DNA from 0.5 g fresh soil using the PowerSoil DNA Isolation Kit (MoBio Laboratories, Carlsbad, CA, USA). DNA extraction was performed according to the manufacturer's instructions. The V3‐V4 region of bacterial 16S rRNA gene was amplified using the primer sets 338F (5′‐ACTCCTACGGGAGGCAGCAG‐3′) and 806R (5′‐GGACTACHVGGGTWTCTAAT‐3′). The ITS1 region of fungi was amplified using the primer sets ITS1‐F (5′‐CTTGGTCATTTAGAGGAAGTAA‐3′) and ITS2 (5′‐TGCGTTCTTCATCGATGC‐3′) (Liu et al., 2021). Amplicons were sequenced on the Illumina MiSeq PE300 platform. The detailed amplicon analysis procedure has been described in a previous study by Liu et al., (2021). In brief, after removing low‐quality bases, we assembled the pair‐end reads using FLASH software (Magoč & Salzberg, 2011), and then removed barcodes, primers, and low‐quality reads from the assembled sequences using MOTHUR software (Schloss et al., 2009). We clustered the remaining high‐quality sequences into operational taxonomic units (OTUs) at 97% similarity (Edgar, 2013) and classified each OTU using the Ribosomal Database Project (RDP) Classifier against the Silva 128 database (Q. Wang et al., 2007) for bacteria and UNITE database (Abarenkov et al., 2010) for fungi. We rarefied each sample to 18,877 reads for bacteria and 24,945 for fungi to ensure equal sequencing depth.

### 2.4 Calculation of species turnover and nestedness

To assess the mechanisms of alterations in community assemblage under N input, we quantified species turnover and nestedness components according to the method proposed by Baselga (2010). This method partitioned the pairwise Sørensen dissimilarity index (*β*_sor_) into two additive components: the fraction of dissimilarity due to species turnover (*β*_sim_) plus the fraction of dissimilarity due to nestedness (*β*_nes_) (Baselga, 2010). We calculated pairwise Sørensen dissimilarly to evaluate dissimilarity in community composition between each N treatment and the ambient control in each block, respectively (Figure S1). The Sørensen dissimilarity is formulated as follows:

$$\beta_{\mathrm{sor}}=\frac{\left(b+c\right)}{\left(2a+b+c\right)}$$

where *a* is the number of species shared in both the N addition and the ambient N plots in the same block; *b* and *c* are the numbers of species that uniquely occur in the two sites, respectively.

The *β*_sim_ is calculated as follows:

$$\beta_{\mathrm{sim}}=\frac{\left(\min\;\left(b,c\right)\right)}{\left(a+\min\left(b,c\right)\right)}$$

Finally, *β*_nes_ is calculated by *β*_sor_ − *β*_sim_.

To promote understanding of nestedness and turnover of community assembly, we quantified the absent resident species and the colonizing species under each N addition. Specifically, we defined all species in all 4 ambient N replicates as local species pools. Comparing with the species pool, we quantified the number of species that were absent in one, two, three, and all of the four replicated plots. If the species was absent in all of the four replicate plots, we defined it as species loss. Correspondingly, we also quantified the number of new species which occurred in one, two, three, and all of the four replicate plots. If the new species was present in all of the four replicate plots, we defined it as new species occurring.

### 2.5 Statistical analyses

We performed permutational multivariate analysis of variance (PERMANOVA) to assess whether N addition affects the community composition of plants, soil bacteria, and fungi (Anderson et al., 2006). The responses of plant, soil bacterial, and fungal communities to N addition were visualized by the ordination of nonmetric multidimensional scaling (NMDS) (Oksanen et al., 2011). We also conducted permutational analysis of multivariate dispersions (PERMDISP) to assess the effect of N addition on the homogeneity of community dispersion (Anderson & Walsh, 2013). These analyses were run based on the Sørensen dissimilarly index, which is computed based on species presence/absence matrix and provides values of dissimilarity from 0 (completely similar) to 1 (completely dissimilar). We further compared total Sørensen dissimilarity between each N input and the ambient (*β*‐diversity) of plants, bacteria, and fungi. Then, univariate regressions were used to test the patterns of total Sørensen dissimilarity between N input and the ambient (*β*‐diversity) of plants, bacteria, and fungi with increasing N input. We also compared the slopes of the three linear functions among plants, bacteria, and fungi using covariance analysis.

We conducted multi‐model averaging based on second‐order Akaike's Information Criterion (AICc) to evaluate changes in environmental variables for the explanation of turnover and nestedness components of plant, bacterial, and fungal community assembly, respectively. Soil total N and DIN were chosen as N availability indicators. Soil pH was chosen to represent soil acidification. Exchangeable Al^3+^ and Mn^2+^ were chosen as indicators of soil biogeochemistry. We calculated changes in these variables between each N treatment plot and the ambient N plot in each block to be environmental indicators in models. Before analysis, we checked multicollinearity among predictors by their variance inflation factors (VIF). All the predictors were retained due to the relatively low VIF of 1.9–5.0. Model averaging was performed using selected multiple models based on a threshold of ΔAICc<4. During analysis, we standardized all predictors to interpret parameter estimates. Since predictors were all Z‐scored before analyses, the relative effect of each predictor can be simply calculated as the ratio between its parameter estimate and the sum of all parameter estimates and expressed in % (Grueber et al., 2011).

All analyses were performed in the R software (R Development Core Team, 2015). The PERMANOVA, PERMDISP, and NMDS were implemented with the function of *adonis*, *betaperdisp*, and *metaMDS* in the R package *vegan*. The partitioning of dissimilarity was performed using *beta.pair* function in the R package *betapart* (Baselga, 2010). VIF was checked in the R package *car*. Model averaging was performed using the “*dredge*” function in the R package *MuMin* (Barton, 2013).

## 3 RESULTS

### 3.1 Differences in plant, bacterial, and fungal dissimilarity with increasing N addition levels

**FIGURE 1:**
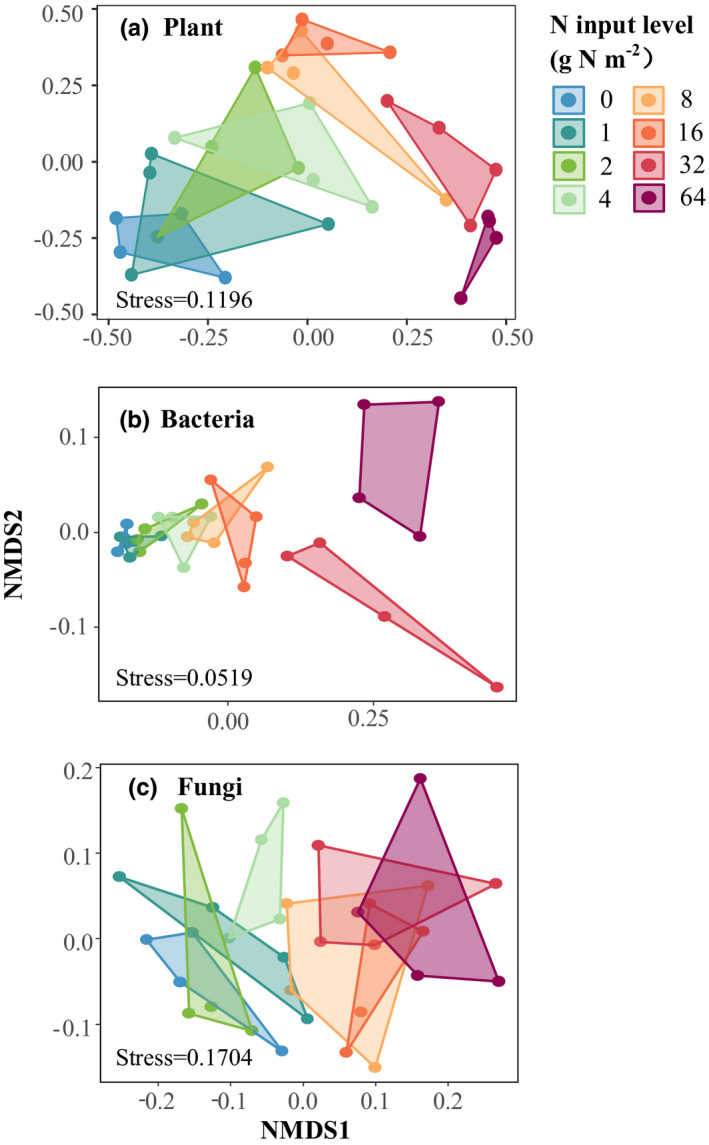
Two‐dimensional nonmetric multidimensional scaling (NMDS) ordination based on Sørensen dissimilarly displaying differences in community composition of plants (a), soil bacteria (b), and fungi (c) in response to N input

Plant (PERMANOVA, *F* = 4.557, *R* ^2^ = .571, *p* = .001; PERMDISP2, *F* = 0.190, *p* = .984), bacterial (PERMANOVA, *F* = 6.222, *R* ^2^ = .645, *p* = .001; PERMDISP2, *F* = 2.050, *p* = .079), and fungal (PERMANOVA, *F* = 2.710, *R* ^2^ = .442, *p* = .001; PERMDISP2, *F* = 0.465, *p* = .834) community composition were all significantly affected by N input (Figure 1).

**FIGURE 2:**
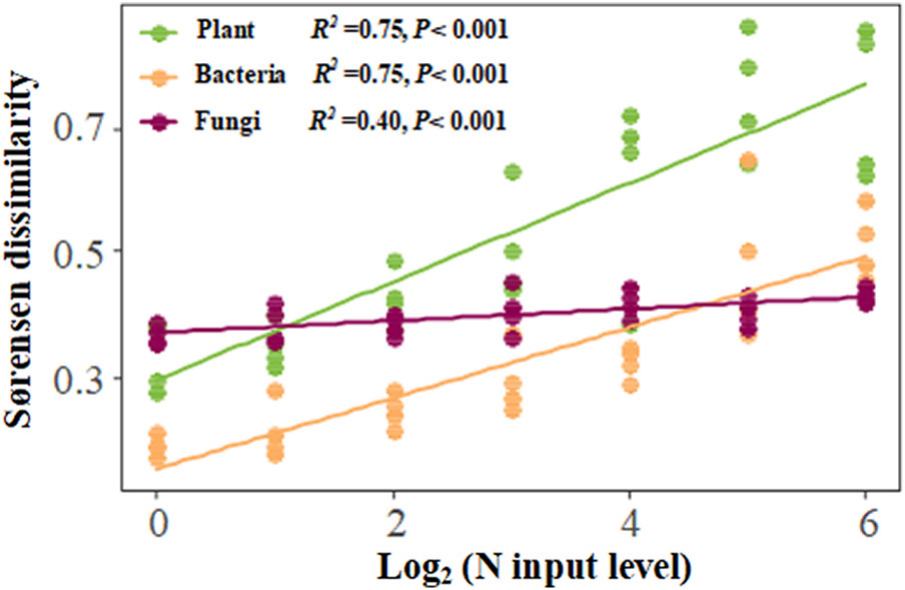
The relationships between total beta‐diversity measured as Sørensen dissimilarity and N input level. The Sørensen dissimilarity indices were calculated between each N input treatment and the ambient. The values of *x*‐axis mean logarithm of N input level to base 2. Significant linear regression lines are shown (*p* < .05)

The magnitude of N‐induced dissimilarity of plant and bacterial and fungal communities all linearly increased with increasing N input (Figure 2). Plant and bacterial communities were more sensitive to N input than fungi (Slope test: both *p* < .001). Fungal dissimilarity remained within a narrow range across the N gradient.

### 3.2 Species turnover and nestedness components

**FIGURE 3:**
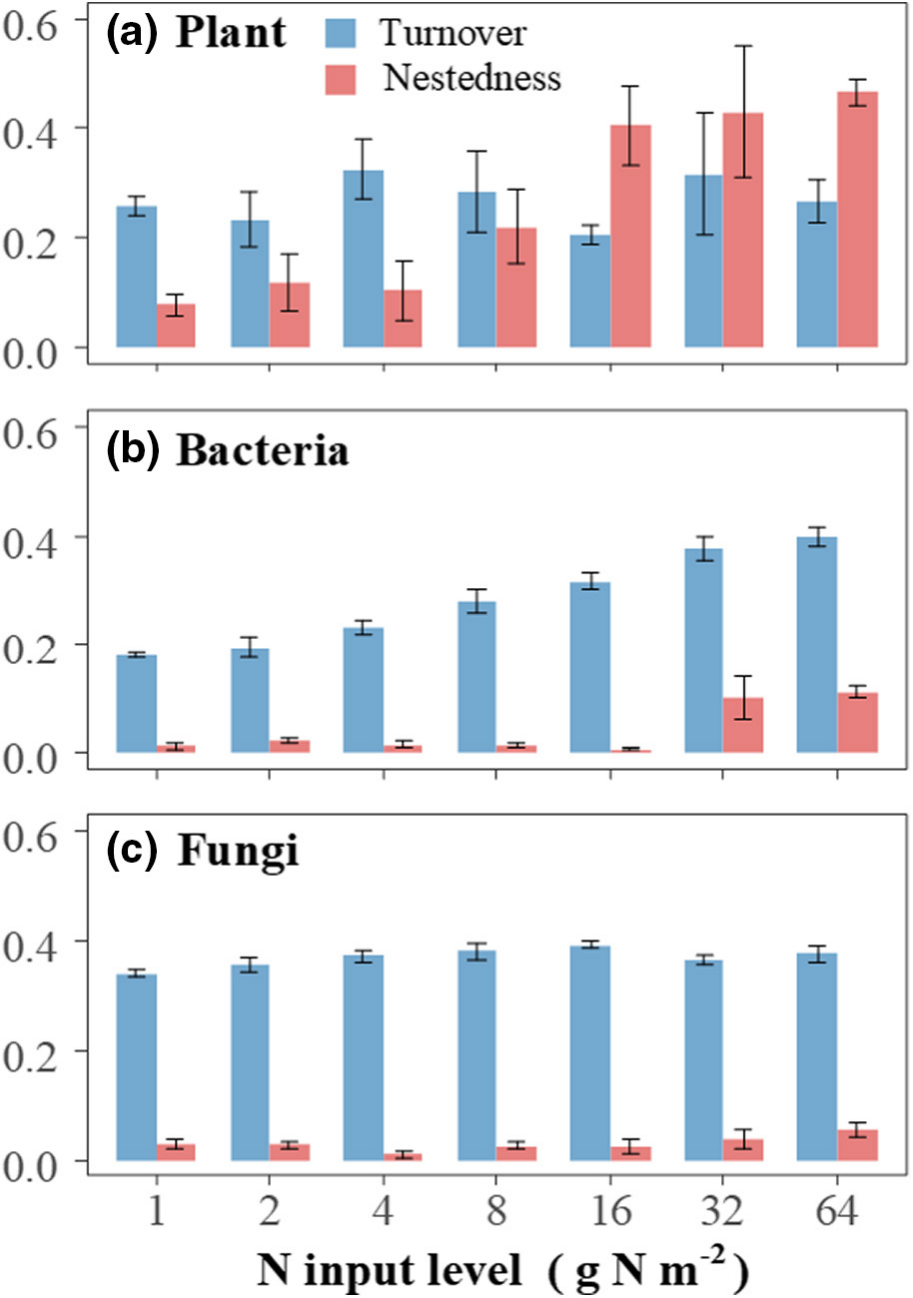
Species turnover and nestedness components of community dissimilarity of plants (a), soil bacteria (b), and fungi (c) between each N input treatment and the ambient treatment, respectively. Each bar represents the mean (± SE, *n* = 4) values for each N input treatment

Turnover and nestedness components of plants, soil bacteria, and fungi showed divergent responses to N input. Specifically, the turnover component of plant dissimilarity was greater than nestedness under lower N input, whereas nestedness was more pronounced than turnover under higher N input (Figure 3a). The turnover component of bacterial dissimilarity was much larger than the nestedness component (Figure 3b) across the N gradient. However, the nestedness sharply increased at the level of 32 g N m^−2^ input. Different from plants and bacteria, the turnover component was greater than nestedness in the dissimilarity of fungal communities and remained relatively stable across the N gradient (Figure 3c).

### 3.3 Environmental factors in driving turnover and nestedness components

**FIGURE 4:**
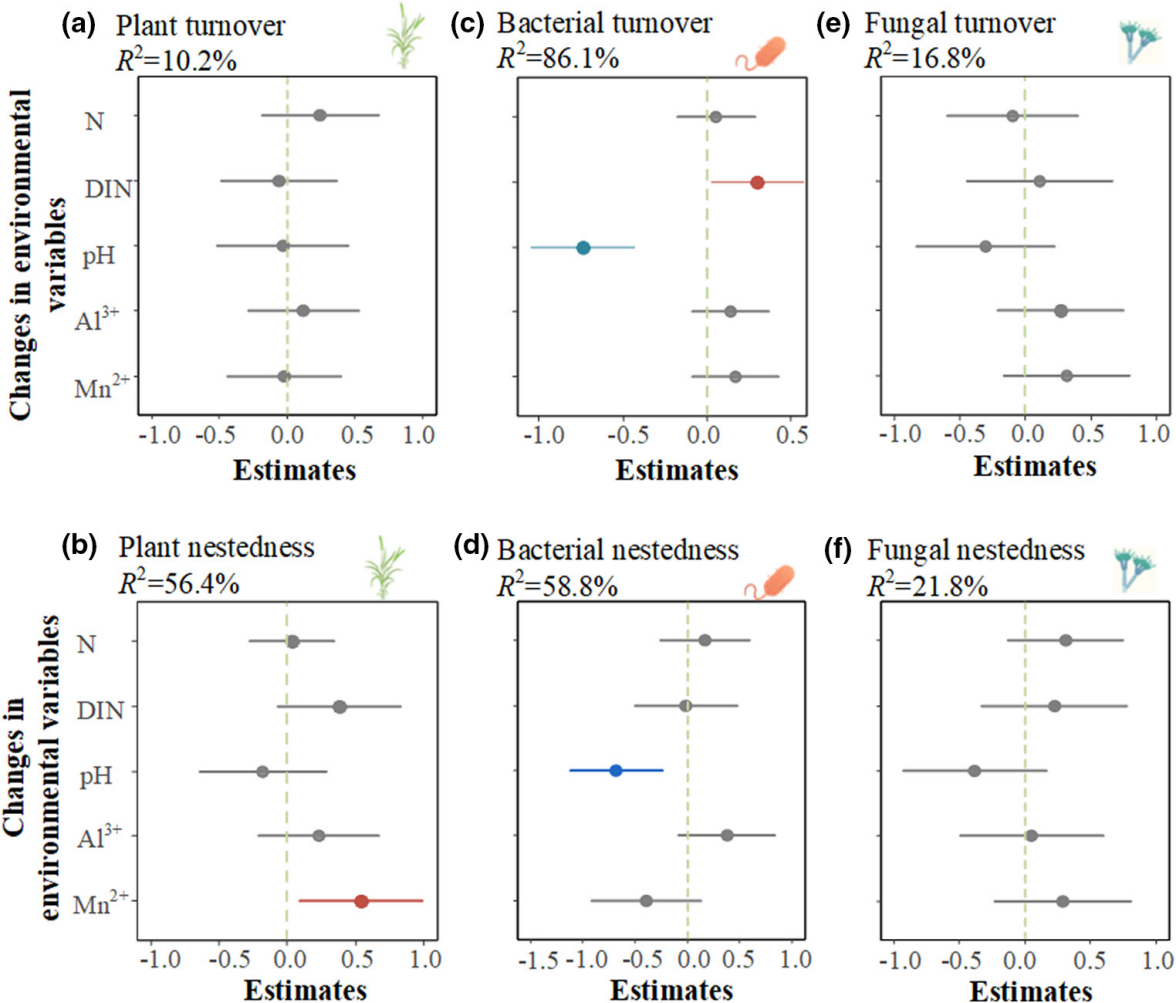
Effects of environmental variables on plant nestedness (a, b), bacterial (c, d), and fungal (e, f) turnover. The average parameter estimate (standardized regression coefficients) of the model predictors and their associated 95% confidence intervals are shown. The red and blue points represent the significant positive and negative predictors in the models. N: Soil total nitrogen; DIN: Soil dissolved inorganic nitrogen

The results of the model averaging analysis showed that changes in environmental factors explained 56.4% of the variability of plant nestedness and soil extractable Mn^2+^ concentration was the best predictor, while plant turnover was not significantly related to environmental changes (Figure 4a and b). N‐induced environmental changes explained 86.1% of the variability of soil bacterial taxa turnover (Figure 4c) with both soil pH and increased N availability being the best predictors. Similarly, soil bacterial pH was also the best predictor for bacterial nestedness (Figure 4d). However, environmental changes had no significant impact on soil fungal taxa turnover and nestedness in the model (Figure 4e and f).

## 4 DISCUSSION

**FIGURE 5:**
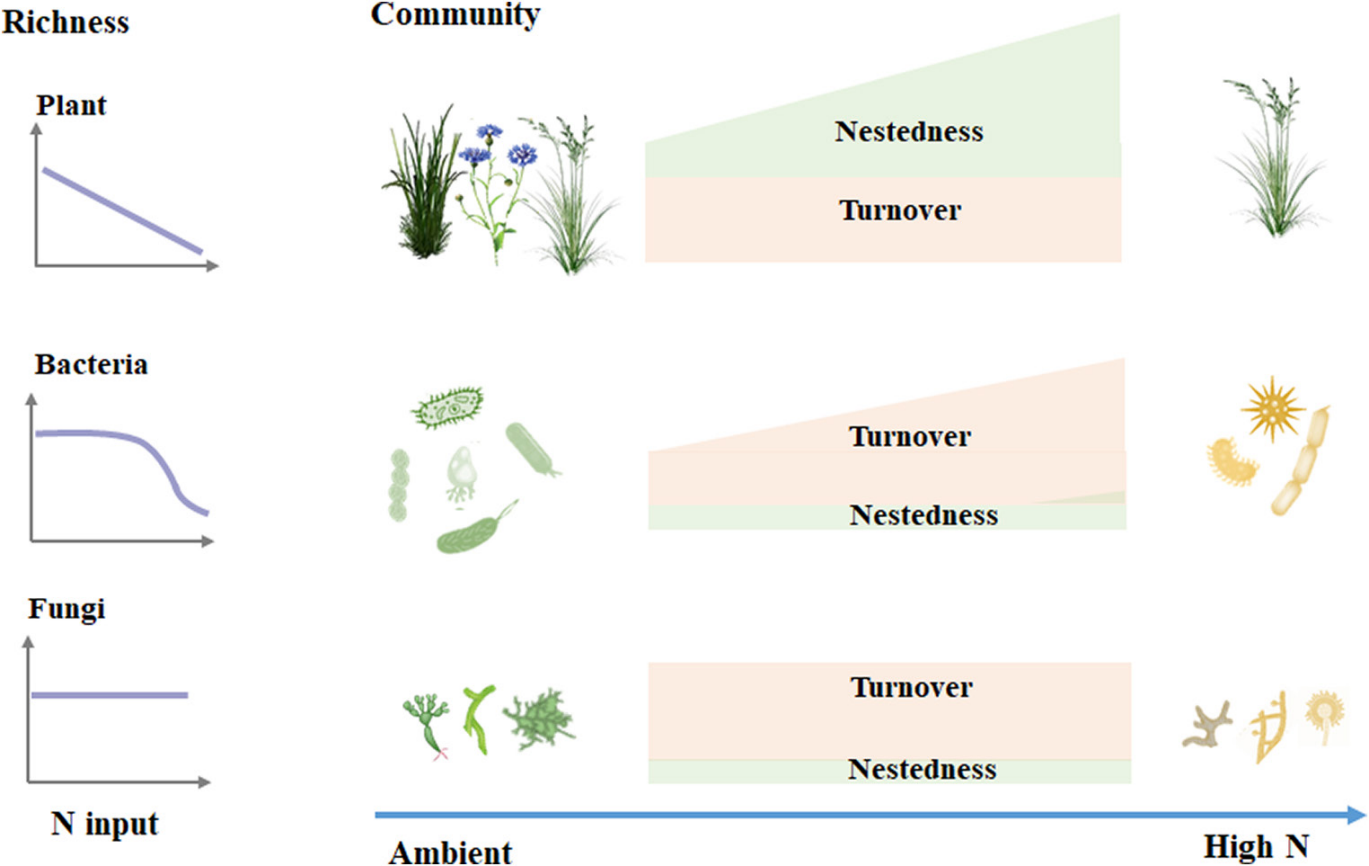
A conceptual figure showing species turnover and nestedness processes driving shifts in community composition of plants, soil bacteria, and fungi along an N input gradient in a semiarid steppe

By quantifying species turnover and nestedness of the *β*‐diversity of plants and soil microbes with increasing N addition, we unveiled the differential mechanisms of plant and soil microbial community assembly under N enrichment (Figure 5). Specifically, N input significantly altered the community structure of plants, soil bacteria, and fungi. Community dissimilarity of plant and bacteria caused by N input was both more sensitive to N input amount than that of fungi. Changes in plant communities were predominantly driven by species turnover under lower N input but by nestedness under higher N input, whereas the differences in soil bacterial and fungal communities were both mainly due to taxa turnover under N input. The proportion of turnover increased for bacterial but remained relatively constant for fungal communities as N input increased.

One previous study has found that plant species richness declined with increasing N input (Liu et al., 2021). In this study, the increased nestedness further pointed out that plants under higher N input tended to be subsets of plants that existed under the ambient and lower N input, which was also reflected by the increasing plant species loss with increasing N input (Figure S2). The increasing nestedness of plant communities is consistent with some previous reports that N enrichment reduces plant richness through local extinction of native species in grasslands (Dupre et al., 2010; Hodapp et al., 2018). The pattern of increasing nestedness component could be attributed to the intensified environmental filtering (Borer et al., 2014; Harpole & Tilman, 2007; Hautier et al., 2009). Supporting this speculation, we found that increased soil‐exchangeable Mn^2+^ concentration (Figure S3a) was the best predictor for nestedness in plant communities induced by N enrichment (Figures 4b and S4a). Indeed, forbs have been reported to be less tolerant of increasing Mn^2+^ toxicity and consequently were progressively filtered out with increasing N input in this same experiment (Bai et al., 2015; Tian et al., 2016; Tian et al., 2020). This increasing progressive filter could create nestedness. Additionally, high N input could intensify interspecies competition for light and facilitate taller species (Hautier et al., 2009). Plant communities were dominated by taller plant species such as *Leymus chinensis* rather than shorter plants such as *Artemisia frigida* and *Chinese Cleistogenes* in one previous study by Liu et al., (2021), contributing to the increasing nestedness pattern. This increasing nestedness of plants reflects the orderly manner of community assembly (Ulrich et al., 2009), further pointing to the role of environmental selection (Liu et al., 2021) and interspecific competition in shaping plant communities under N enrichment. However, the increase in nestedness of plants would promote biotic homogenization, resulting in loss of diversity at a regional scale (Baeten et al., 2012; de Castro Solar et al., 2015). Note that the nestedness was lower than turnover under lower N additions, indicating that some plants in this region may adapt to low N input and native plant community structure may be maintained through the colonization of such new species (Figure S2a).

We expected that changes in microbial communities would exhibit the same phenomenon following plant communities because microbial populations highly depend on the resources provided by plants (de Souza et al., 2015; van der Putten et al., 2013). However, changes in bacterial and fungal communities were both mainly driven by taxa turnover, rather than taxa nestedness (Figures 2 and 3), further revealing the different ways of community re‐organization between plants and soil microbes under N enrichment. This provided one explanation for some previous results reporting that N addition changed bacterial (Fierer et al., 2012; Ramirez et al., 2010) and fungal (Mueller et al., 2015) communities but did not affect their richness.

We found that N availability and soil pH both explained the turnover of bacterial communities in response to N enrichment (Figure 4c). On the one hand, low‐resource‐adapted bacterial groups can be outcompeted by copiotrophic groups in nutrient‐rich environments (Fierer et al., 2007; Leff et al., 2015; Morrissey et al., 2017). Accordingly, increased N availability‐induced shifts in bacterial communities toward copiotrophic taxa in this experiment (Liu et al., 2020) support the increasing taxa turnover of bacterial communities. On the other hand, microbial taxa differ in acid tolerance (Rousk et al., 2010). Consequently, N‐induced soil acidification (Figures S3b and S4b) would promote bacterial turnover to favor species adapted to lower pH. Indeed, we found the relative abundance of *Bacteroidetes* increased with intensified soil acidification (Liu et al., 2020). However, the relative abundance of *Actinobacteria* and *Chloroflexi* preferring neutral pH conditions (Wang et al., 2019) decreased. Note that the nestedness component increased when N input exceeded 16 g N m^−2^ (Figure 3). This implies that the extreme soil acidification above 16 g N m^−2^ may sharply exclude some resident bacterial taxa with less acid tolerance, reducing bacterial richness and promoting nestedness (Rousk et al., 2010) (Figures 4d and S4c). The increase in nestedness component accompanying species loss demonstrates that nestedness originates from species loss in community assembly (Ulrich et al., 2009).

Fungal community assembly was mainly generated by taxa turnover rather than nestedness under N enrichment. The result of model averaging implies that there was no significant impact of environmental changes on either turnover or nestedness of fungal communities (Figure 4e and f), consistent with some previous reports that fungal communities were mainly governed by stochastic processes at local scales (Zheng et al., 2021) and not related to local environmental factors (Li et al., 2020; Powell et al., 2015; Wang et al., 2019) nor roots of host plants (Beck et al., 2015). The inherent traits of filamentous fungi form are responsible for this dominant turnover of fungal communities (de Vries et al., 2018). The fungal mycelial structure is beneficial for occupying more niches regardless of environmental conditions, thus favoring species turnover. The relatively constant turnover and negligible nestedness further provide the underlying mechanisms for the unchanged fungal richness under N enrichment reported in one previous work (Liu et al., 2021). Overall, this finding suggests that fungal richness could remain stable under N enrichment, but this apparent stability is often accompanied by the marked turnover of taxa (Figure 5c).

## 5 CONCLUSION

This study firstly elucidated the differential mechanisms of re‐structuring plant and microbial communities by two different pathways of turnover and nestedness in response to N enrichment. Increasing nestedness contributed to plant community dissimilarity with increasing N input, whereas dissimilarity of bacterial communities was largely driven by increasing taxa replacement. The increasing nestedness of plant communities as well as increasing turnover of bacterial communities were both due to strong environmental filtering caused by increasing N input. However, the turnover of fungal communities remained relatively stable with increasing N input. This study provides a clearer picture of how plant and soil microbial population assembly respond to extra N input and further implies the distinct mechanisms that underlie plant and microbial community assembly with N enrichment. Caution is needed in extrapolating the results to other ecosystems or global scales since we only focused on semiarid grassland. Furthermore, we recognize that using NH_4_^+^‐N and NO_3_^+^‐N as estimated parameters could affect the interpretation of the results because plants and soil microorganisms could directly immobilize NH_4_^+^‐N and NO_3_^+^‐N differently. Nevertheless, our findings suggest an approach to exploring community assembly mechanism between plants and soil microbes under N enrichment. Further research is required to evaluate how functional *β*‐diversity responds to N enrichment and the contribution of turnover and nestedness‐resultant components to functional *β*‐diversity, advancing the understanding of ecosystem functions.

## AUTHOR CONTRIBUTIONS

**Weixing Liu:** Conceptualization (lead); data curation (equal); formal analysis (equal); funding acquisition (lead); project administration (lead); writing – original draft (lead). **Xian Yang:** Formal analysis (equal); methodology (equal); writing – original draft (lead). **Lin Jiang:** Formal analysis (equal); writing – original draft (supporting). **Lulu Guo:** Visualization (equal); writing – original draft (supporting). **Yaru Chen:** Visualization (supporting); writing – original draft (supporting). **Sen Yang:** Investigation (equal). **LingLi Liu:** Conceptualization (lead); project administration (lead).

## CONFLICT OF INTEREST

The authors declare that they have no competing interests.

## ETHICAL APPROVAL

Compliance with ethical standards.

## Supporting information


Figure S1–S4

## Data Availability

DNA sequences: NCBI SRA: PRJNA573484; PRJNA573488.
