# Supplementary material for: Partitioning of beta‐diversity reveals distinct assembly mechanisms of plant and soil microbial communities in response to nitrogen enrichment
Source: Ecol Evol. 2022 Jun 17;12(6):e9016. doi: 10.1002/ece3.9016 (PMC9205676; doi:10.1002/ece3.9016)
Supplement: Supplementary file 1 — Figure S1–S4 [file ECE3-12-e9016-s001.docx]

**Supporting information.**

**Partitioning of beta-diversity reveals distinct assembly mechanisms of plant and soil microbial communities in response to nitrogen enrichment**

**This file includes:**

Figures S1, S2, S3 and S4

Figure S1. Picture of field experiment (a) and layout of experimental setup (b). The plots within red block were investigated and sampled in this study.


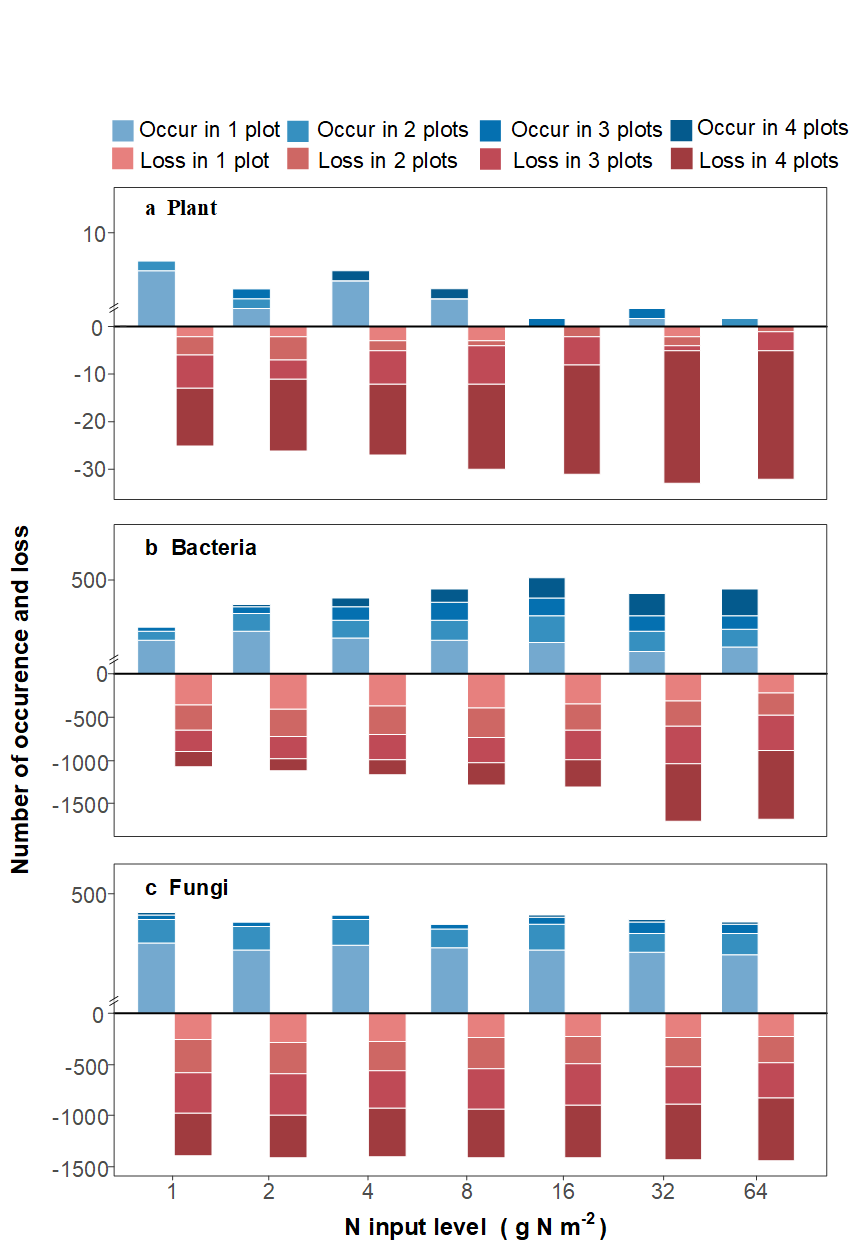


Figure S2. The number of existence and loss of plant species (a), bacterial (b) and (c) fungal taxa under each N input treatment when compared with the total ambient species pool. Occur in 1, 2, 3, and 4 plots mean the number of the species which as new occurrence in 1, 2, 3, and 4 replicated plots under each N input treatment comparing to the whole 4 control plots, respectively. Loss 1, 2, 3, and 4 mean the number of the species which disappears in the 1, 2, 3, and 4 replicated plots under N input treatment comparing to the whole 4 control plots, respectively.

Figure S3. Effects of N input on soil extractable Mn^2+^ concentration (a) and soil pH value (b).


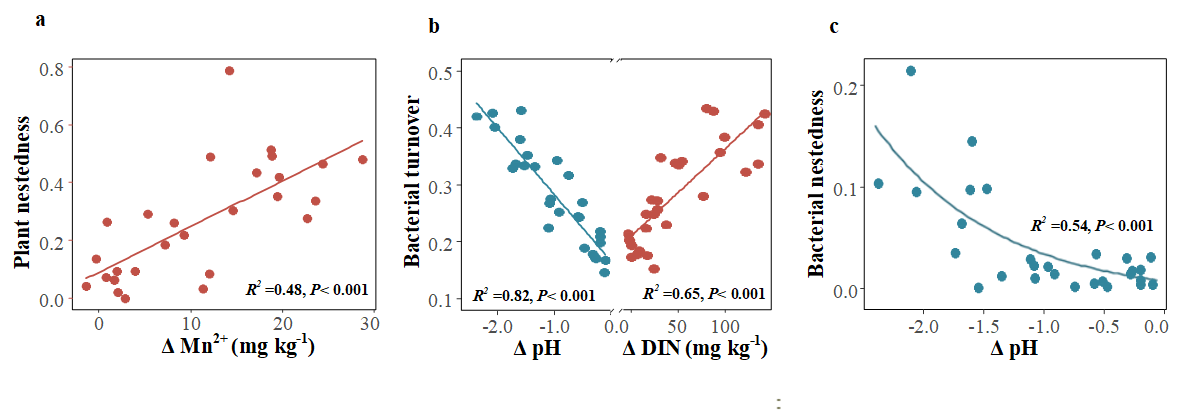


Figure S4. Effects of changes in soil extractable Mn^2+^ concentration on plant nestedness (a), changes in soil pH value and DIN on bacterial turnover (b), and changes in pH value on bacterial nestedness (c) with increasing N input gradient. DIN: soil dissolved inorganic nitrogen.
